# On the *x*-coordinates of Pell equations that are sums of two Padovan numbers

**DOI:** 10.1007/s40590-021-00312-8

**Published:** 2021-02-23

**Authors:** Mahadi Ddamulira

**Affiliations:** 1 Institute of Analysis and Number Theory, Graz University of Technology, Kopernikusgasse 24/II, 8010 Graz, Austria; 2 Max Planck Institute for Software Systems, Saarland Informatics Campus, Campus E1 5, 66123 Saarbrücken, Germany; 3 Department of Mathematics, Makerere University, P.O. Box 7062, Kampala, Uganda

**Keywords:** Padovan number, Pell equation, Linear form in logarithms, Reduction method, Primary 11B39, 11D45, Secondary 11D61, 11J86

## Abstract

Let $$ (P_{n})_{n\ge 0} $$ be the sequence of Padovan numbers defined by $$ P_0=0 $$, $$ P_1 = P_2=1$$, and $$ P_{n+3}= P_{n+1} +P_n$$ for all $$ n\ge 0 $$. In this paper, we find all positive square-free integers *d* such that the Pell equations $$ x^2-dy^2 = N $$ with $$ N\in \{\pm 1, \pm 4\} $$, have at least two positive integer solutions (*x*, *y*) and $$(x^{\prime }, y^{\prime })$$ such that both *x* and $$x^{\prime }$$ are sums of two Padovan numbers.

## Introduction

Let $(P_{n})_{n\ge 0}$ be the sequence of Padovan numbers defined by the linear recurrence

$$\begin{aligned} P_0=0, \,P_1= 1, \,P_2=1, \text { and } P_{n+3}= P_{n+1}+P_n \text { for all } n\ge 0. \end{aligned}$$

The Padovan sequence appears as sequence *A*000931 on the On-Line Encyclopedia of Integer Sequences (OEIS) [20]. The first few terms of this sequence are

$$\begin{aligned} 0, 1, 1, 1, 2, 2, 3, 4, 5, 7, 9, 12, 16, 21, 28, 37, 49, 65, 86, 114, 151, 200, 265, 351 \ldots . \end{aligned}$$

Let $d\ge 2$ be a positive square-free integer. It is well known that the Pell equations

1 $$\begin{aligned} x^{2}-dy^{2}=\pm 1, \end{aligned}$$

and

2 $$\begin{aligned} X^{2}-dY^{2}=\pm 4, \end{aligned}$$

have infinitely many positive integer solutions (*x*, *y*) and (*X*, *Y*) , respectively. By putting $(x_1, y_1)$ and $(X_1, Y_1)$ for the smallest positive solutions to (1) and (2), respectively, all the solutions $(x_k, y_k)$ and $(X_k, Y_k)$ have the form

$$\begin{aligned} x_k+y_k\sqrt{d} = (x_1+y_1\sqrt{d})^k\qquad {\mathrm{for\,all}} \quad k\in {\mathbb {Z}}^{+}, \end{aligned}$$

and

$$\begin{aligned} \dfrac{X_k+Y_k\sqrt{d}}{2} = \left( \dfrac{X_1+Y_1\sqrt{d}}{2}\right) ^k\qquad {\mathrm{for\,all}} \quad k\in {\mathbb {Z}}^{+}. \end{aligned}$$

Furthermore, $(x_k)_{k\ge 1}$ and $(X_k)_{k\ge 1}$ are binary recurrent sequences. More exactly, the following formulae

3 $$\begin{aligned} x_k=\dfrac{(x_1+y_1\sqrt{d})^{k}+(x_1-y_1\sqrt{d})^{k}}{2}, \end{aligned}$$

and

4 $$\begin{aligned} X_k = \left( \dfrac{X_1+Y_1\sqrt{d}}{2}\right) ^{k}+ \left( \dfrac{X_1-Y_1\sqrt{d}}{2}\right) ^{k} \end{aligned}$$

hold for all positive integers *k*.

In the recent years, Luca et al. [17] considered the Diophantine equation

5 $$\begin{aligned} x_{k}=T_n, \end{aligned}$$

where $x_k$ is given by (3) and $(T_n)_{n \ge 0}$ is the Tribonacci sequence defined by $T_0 = 0$, $T_1 =T_2= 1$, and $T_{n+3} = T_{n+2} + T_{n+1}+T_{n}$ for all $n \ge 0$. The Tribonacci sequence appears as sequence *A*000073 on the OEIS [20]. The authors in [17] proved that Eq. (5) has at most one solution (*k*, *n*) in positive integers for all *d* except for $d=2$ when Eq. (5) has the three solutions $(k,n)=\{(1,1), (1,2), (3,5)\}$ and when $d=3$ case in which Eq. (5) has the two solutions $(k,n)=\{(1,3), (2,5)\}$.

Inspired by the main result of Luca et al. [17], E. F. Bravo et al. [3, 4] studied the Diophantine equation

6 $$\begin{aligned} x_k = T_m+T_n. \end{aligned}$$

They proved that for each square-free integer $d\ge 2$, there is at most one positive integer *k* such that $x_k$ admits the representation (6) for some nonnegative integers $0\le m\le n$, except for $d\in \{2,3,5,15,26\}$. Furthermore, they explicitly stated all the solutions for these exceptional cases.

In the same spirit of the main result of Luca et al. [17], Rihane et al. [21] studied the Diophantine equations

7 $$\begin{aligned} x_k = P_n \qquad \text {and}\qquad X_k=P_n, \end{aligned}$$

where $x_k$ and $X_k$ are given by (3) and (4), respectively. They proved that for each square-free integer $d\ge 2$, there is at most one positive integer *x* participating in the Pell equation (1) and at most one positive integer *X* participating in the Pell equation (2) that is a Padovan number with a few exceptions of *d* that they effectively computed. Furthermore, the exceptional cases were $d\in \{2,3,5,6\}$ and $d=5$ for the the first and second equations in (7), respectively. Several other related problems have been studied where $x_k$ belongs to some interesting positive integer sequences. For example, see [8, 9, 11, 12, 14–16, 18].

## Main results

In this paper, we study the same problem considered by E. F. Bravo et al.[3, 4] but with Padovan numbers instead of Tribonacci numbers. We also extend the results from the Pell equation (1) to the Pell equation (2). In both cases we find that there are only finitely many solutions that we effectively compute. Since $$ P_1=P_2=P_3 = 1$$, we discard the situations when $$ n=1 $$ and $$ n=2 $$ and just count the solutions for $$ n=3 $$. Similarly, $$ P_4=P_5=2 $$, so we just count the solutions for $$ n=5 $$.

The main aim of this paper is to prove the following results.

### Theorem 1

*For each square-free integer* $d\ge 2$, *there is at most one positive integer* *k* *such that*

8 $$\begin{aligned} x_k=P_n+P_m \end{aligned}$$

*except when* $d\in \{2,3,6,15,110,483\}$ *in the* $+1$ *case and* $d\in \{2,5,10,17\}$ *in the* $-1$ *case.*

### Theorem 2

*For each square-free integer* $d\ge 2$, *there is at most one positive integer* *k* *such that*

9 $$\begin{aligned} X_k=P_n+P_m \end{aligned}$$

*except when* $d\in \{3,5,21\}$ *in the* $+4$ *case and* $d\in \{2, 5\}$ in the $-4$ case.

For the exceptional values of *d* listed in Theorem 1 and Theorem 2, all solutions (*k*, *n*, *m*) are listed at the end of the proof of each result. The main tools used in this paper are lower bounds for nonzero linear forms in logarithms of algebraic numbers “á la Baker” and the Baker-Davenport reduction procedure, as well as the elementary properties of the Padovan sequence and solutions to Pell equations. Computations are done with the help of a computer program in *Mathematica*.

## Preliminary results

### The Padovan sequence

Here, we recall some important properties of the Padovan sequence $(P_n)_{n\ge 0}$. The characteristic equation

$$\begin{aligned} x^3-x-1 = 0, \end{aligned}$$

has roots $\alpha , \beta , \gamma = {\bar{\beta }}$, where

10 $$\begin{aligned} \alpha =\dfrac{r_1+r_2}{6}, \qquad \beta = \dfrac{-(r_1+r_2)+\sqrt{-3}(r_1-r_2)}{12}, \end{aligned}$$

with

11 $$\begin{aligned} r_1=\root 3 \of {108+12\sqrt{69}} \quad \text {and}\quad r_2=\root 3 \of {108-12\sqrt{69}}. \end{aligned}$$

Furthermore, a Binet-like formula for Padovan numbers is given by

12 $$\begin{aligned} P_n = a\alpha ^{n}+b\beta ^{n}+c\gamma ^{n} \qquad \text { for all} \quad n\ge 0, \end{aligned}$$

where

13 $$\begin{aligned} \quad a=\dfrac{\alpha +1}{(\alpha -\beta )(\alpha -\gamma )}, \quad b= \dfrac{\beta +1}{(\beta -\alpha )(\beta -\gamma )}, \quad c = \dfrac{\gamma +1}{(\gamma -\alpha )(\gamma -\beta )}={\bar{b}}. \end{aligned}$$

Numerically, the following estimates hold:

14 $$\begin{aligned} \begin{aligned} 1.32&<\alpha<1.33,\\ 0.86&< |\beta |=|\gamma |=\alpha ^{-\frac{1}{2}}< 0.87,\\ 0.54&<a<0.55,\\ 0.28&<|b|=|c|<0.29. \end{aligned} \end{aligned}$$

From (10), (11), and (14), it is easy to see that the contribution of the complex conjugate roots $\beta$ and $\gamma$, to the right-hand side of (12), is very small. More exactly, setting $e(n):=P_n-a\alpha ^{n}$ and taking into account the facts that $|\beta |=|\gamma |=\alpha ^{-\frac{1}{2}}$ and $|b|=|c|< 0.29$ (by (14)), it follows that, for any $n\ge 1$,

15 $$\begin{aligned} |e(n)|=\left| b\beta ^{n}+c\gamma ^{n}\right| \le |b||\beta |^{n}+ |c||\gamma |^{n}= |b|\alpha ^{-\frac{n}{2}}+ |c| \alpha ^{-\frac{n}{2}}< 2\cdot 0.29 \cdot \alpha ^{-\frac{n}{2}} < \dfrac{1}{\alpha ^{n/2}}. \end{aligned}$$

Finally, one can prove by induction that

16 $$\begin{aligned} \alpha ^{n-2}\le P_n \le \alpha ^{n-1} \quad \text {holds for all }\quad n\ge 4. \end{aligned}$$

### Linear forms in logarithms

Let $\eta$ be an algebraic number of degree *d* with minimal primitive polynomial over the integers

$$\begin{aligned} a_{0}x^{d}+ a_{1}x^{d-1}+\cdots +a_{d} = a_{0}\prod _{i=1}^{d}(x-\eta ^{(i)}), \end{aligned}$$

where the leading coefficient $a_{0}$ is positive and the $\eta ^{(i)}$’s are the conjugates of $\eta$. Then, the *logarithmic height* of $\eta$ is given by

$$\begin{aligned} h(\eta ) := \dfrac{1}{d}\left( \log a_{0} + \sum _{i=1}^{d}\log \left( \max \{|\eta ^{(i)}|, 1\}\right) \right) . \end{aligned}$$

In particular, if $\eta = p/q$ is a rational number with $\gcd (p,q) = 1$ and $q>0$, then $h(\eta ) = \log \max \{|p|, q\}$. The following are some of the properties of the logarithmic height function $h(\cdot )$, which will be used in the next sections of this paper without reference:

$$\begin{aligned} \begin{aligned} h(\eta _1\pm \eta _2)&\le h(\eta _1) +h(\eta _2) +\log 2,\\ h(\eta _1\eta _2^{\pm 1})&\le h(\eta _1) + h(\eta _2),\\ h(\eta ^{s})&= |s|h(\eta ) \quad (s\in {\mathbb {Z}}). \end{aligned} \end{aligned}$$

We recall the result of Bugeaud et al. (see [6], Theorem 9.4), which is a modified version of the result of Matveev [19], which is one of our main tools in this paper.

#### Theorem 3

(Matveev according to Bugeaud et al., [6, 19]) *Let* $\eta _1,\ldots ,\eta _t$ *be nonzero elements of an algebraic number field* ${\mathbb {K}} \subset {\mathbb {R}}$ *of degree* $D_{\mathbb {K}}$ *over* ${\mathbb {Q}}$, $b_1,\ldots ,b_t$ *be nonzero integers, and assume that*

$$\begin{aligned} \Lambda :=\eta _1^{b_1}\cdots \eta _t^{b_t} - 1, \end{aligned}$$

*is nonzero. Then*

$$\begin{aligned} \log |\Lambda | > -1.4\times 30^{t+3}\times t^{4.5}\times D_{\mathbb {K}}^{2}(1+\log D_{{\mathbb {K}}})(1+\log B)A_1\cdots A_t, \end{aligned}$$

*where*

$$\begin{aligned} B\ge \max \{|b_1|, \ldots , |b_t|\}, \end{aligned}$$

*and*

$$\begin{aligned} A_i \ge \max \{D_{{\mathbb {K}}} h(\eta _i), |\log \eta _i|, 0.16\},\qquad {\text {for all}}\qquad i=1,\ldots ,t. \end{aligned}$$

### Reduction procedure

During the calculations, we get upper bounds on our variables which are too large, thus we need to reduce them. To do so, we use some results from the theory of continued fractions.

For the treatment of linear forms homogeneous in two integer variables, we use the following well-known classical result in the theory of Diophantine approximation. For further details, we refer the reader to the books of Baker and Wüstholz [2] and Cohen [7].

#### Lemma 1

(Legendre, [2, 7]). *Let* $\tau$ *be an irrational number*, ${p_0}/{q_0}, {p_1}/{q_1}, {p_2}/{q_2}, \ldots$ *be all the convergents of the continued fraction expansion of* $\tau$ *and* *M* *be a positive integer. Let* *N* *be a nonnegative integer such that* $q_N> M$. *Then putting* $a(M):=\max \{a_{i}: i=0, 1, 2, \ldots , N\}$, *the inequality*

$$\begin{aligned} \left| \tau - \dfrac{r}{s}\right| > \dfrac{1}{(a(M)+2)s^{2}}, \end{aligned}$$

*holds for all pairs* (*r*, *s*) *of positive integers with* $0<s<M$.

For a nonhomogeneous linear form in two integer variables, we use a slight variation of a result due to Dujella and Pethő (see [10], Lemma 5a) and itself is a generalization of a result of Baker and Davenport [1]. In this paper, we use an immediate variation of the result of Dujella and Pethő [10] due to J.J. Bravo et al. (see [5], Lemma 1). For a real number *X*, we write $\Vert X \Vert := \min \{|X-n|: n\in {\mathbb {Z}}\}$ for the distance from *X* to the nearest integer.

#### Lemma 2

(Dujella, Pethő according to J. J. Bravo et al. [5, 10]) *Let* *M* *be a positive integer,* *p*/*q* *be a convergent of the continued fraction expansion of the irrational number* $\tau$ *such that* $q>6M$, *and* $A,B,\mu$ *be some real numbers with* $A>0$ *and* $B>1$. *Furthermore, let* $\varepsilon : = \Vert \mu q\Vert -M\Vert \tau q\Vert$. *If* $\varepsilon > 0$, *then there is no solution to the inequality*

$$\begin{aligned} 0<|u\tau -v+\mu |<AB^{-w}, \end{aligned}$$

*in positive integers* *u*, *v*,  *and* *w* *with*

$$\begin{aligned} u\le M \quad {\text {and}}\quad w\ge \dfrac{\log (Aq/\varepsilon )}{\log B}. \end{aligned}$$

At various occasions, we need to find a lower bound for linear forms in logarithms with bounded integer coefficients in three and four variables. In this case, we use the LLL algorithm that we describe below. Let $\tau _1, \tau _2, \ldots \tau _t \in {\mathbb {R}}$ and the linear form

$$\begin{aligned} x_1\tau _1+x_2\tau _2+\cdots +x_t\tau _t \quad \text { with } \quad |x_i|\le X_i. \end{aligned}$$

We put $X:=\max \{X_i\}$, $C> (tX)^{t}$ and consider the integer lattice $\Omega$ generated by

$$\begin{aligned} \mathbf{b }_j: = \mathbf{e }_j+\lfloor C\tau _j\rceil \mathbf{e }_t \quad \text { for} \quad 1\le j\le t-1 \quad \text { and} \quad \mathbf{b }_t:=\lfloor C\tau _t\rceil \mathbf{e }_t, \end{aligned}$$

where *C* is a sufficiently large positive constant.

#### Lemma 3

(LLL-algorithm, [7]) *Let* $X_1, X_2, \ldots , X_t$ *be positive integers such that* $X:=\max \{X_i\}$ *and* $C> (tX)^{t}$ *be a fixed sufficiently large constant. With the above notation on the lattice* $\Omega$, *we consider a reduced base* $\{\mathbf{b }_i \}$ *to* $\Omega$ *and its associated Gram-Schmidt orthogonalization base* $\{\mathbf{b }_i^*\}$. *We set*

$$\begin{aligned} c_1:=\max _{1\le i\le t}\dfrac{\Vert \mathbf{b }_1\Vert }{\Vert \mathbf{b }_i^*\Vert }, \quad \theta :=\dfrac{\Vert \mathbf{b }_1\Vert }{c_1}, \quad Q:=\sum _{i=1}^{t-1}X_i^{2}, \quad \text {and} \quad R:=\dfrac{1}{2}\left( 1+ \sum _{i=1}^{t}X_i\right) . \end{aligned}$$

*If the integers* $x_i$ *are such that* $|x_i|\le X_i$, *for* $1\le i \le t$ *and* $\theta ^2\ge Q+R^2$, *then we have*

$$\begin{aligned} \left| \sum _{i=1}^{t}x_i\tau _i\right| \ge \dfrac{\sqrt{\theta ^2-Q}-R}{C}. \end{aligned}$$

For the proof and further details, we refer the reader to the book of Cohen (see [7], Proposition 2.3.20).

Finally, the following Lemma is also useful. It is Lemma 7 in [13].

#### Lemma 4

(Gúzman Sánchez, Luca, [13]) *Let* $r, \, H,$ *and* *L* *be positive real numbers. If* $r\ge 1$, $H>(4r^2)^r$, *and* $H>L/(\log L)^r$, *then*

$$\begin{aligned} L<2^rH(\log H)^r. \end{aligned}$$

## Proof of Theorem 1

Let $(x_1, y_1)$ be the smallest positive integer solution to the Pell equation (1). We put

17 $$\begin{aligned} \delta :=x_1+y_1\sqrt{d} \quad \text {and} \quad \sigma :=x_1-y_1\sqrt{d}. \end{aligned}$$

From which we get that

18 $$\begin{aligned} \delta \cdot \sigma =x_1^2-dy_1^2 =: N, \quad \text {where} \quad N\in \{\pm 1\}. \end{aligned}$$

Then,

19 $$\begin{aligned} x_k = \dfrac{1}{2}(\delta ^k+\sigma ^k). \end{aligned}$$

Since $\delta \ge 1+\sqrt{2}$, it follows that the estimate

20 $$\begin{aligned} \dfrac{\delta ^{k}}{\alpha ^{4}}\le x_k \le \delta ^k \quad \text { holds for all } \quad k\ge 1. \end{aligned}$$

We assume that $(k_1, n_1, m_1)$ and $(k_2, n_2, m_2)$ are triples of integers such that

21 $$\begin{aligned} x_{k_1}=P_{n_1}+P_{m_1} \quad \text {and} \quad x_{k_2}=P_{n_2}+P_{m_2}. \end{aligned}$$

We assume that $1\le k_1 < k_2$. We also assume that $3\le m_i< n_i$ for $i=1,2$. We set $(k,n,m):=(k_i, n_i, m_i)$, for $i=1,2$. Using the inequalities (16) and (20), we get from (21) that

$$\begin{aligned} \dfrac{\delta ^{k}}{\alpha ^{4}}\le x_k=P_n+P_m\le 2\alpha ^{n-1}\quad \text {and} \quad \alpha ^{n-2}\le P_n+P_m = x_k\le \delta ^{k}. \end{aligned}$$

The above inequalities give

$$\begin{aligned} (n-2)\log \alpha<k\log \delta < (n+3)\log \alpha +\log 2. \end{aligned}$$

Dividing through by $\log \alpha$ and setting $c_2:=1/\log \alpha$, we get that

$$\begin{aligned} -2<c_2k\log \delta -n<3+c_2\log 2, \end{aligned}$$

and since $\alpha ^3>2$, we get

22 $$\begin{aligned} |n-c_2k\log \delta |<6. \end{aligned}$$

Furthermore, $k<n$, for if not, we would then get that

$$\begin{aligned} \delta ^{n}\le \delta ^{k}<2\alpha ^{n+3}, \quad \text {implying}\quad \left( \dfrac{\delta }{\alpha }\right) ^{n}<2\alpha ^{3}, \end{aligned}$$

which is false since $\delta \ge 1+\sqrt{2}$, $\alpha \in (1.32, 1.33)$ (by (14)), and $n\ge 4$.

Besides, given that $k_1<k_2$, we have by (16) and (21) that

$$\begin{aligned} \alpha ^{n_1-2}\le P_{n_1}\le P_{n_1}+P_{m_1}=x_{k_1}<x_{k_2}= P_{n_2}+P_{m_2} \le 2P_{n_2}<2\alpha ^{n_2-1}. \end{aligned}$$

Thus, we get that

23 $$\begin{aligned} n_1<n_2+4. \end{aligned}$$

### An inequality for *n* and *k*

Using the Eqs. (8), (12), (19), and (21), we have

$$\begin{aligned} \dfrac{1}{2}(\delta ^{k}+\sigma ^{k})=P_n+P_m=a\alpha ^{n}+e(n)+a\alpha ^{m}+e(m). \end{aligned}$$

Therefore,

$$\begin{aligned} \frac{1}{2}\delta ^{k}-a(\alpha ^{n}+\alpha ^{m})=-\dfrac{1}{2}\sigma ^{k}+e(n)+e(m), \end{aligned}$$

and by (15), we have

$$\begin{aligned} |\delta ^{k}(2a)^{-1}\alpha ^{-n}(1+\alpha ^{m-n})^{-1}-1|&\le \dfrac{1}{2\delta ^{k}a(\alpha ^{n}+\alpha ^{m})}+\dfrac{2|b|}{\alpha ^{n/2}a(\alpha ^{n}+\alpha ^{m})}\\ {}&\quad +\dfrac{2|b|}{\alpha ^{m/2}a(\alpha ^{n}+\alpha ^{m})}\\&\le \dfrac{1}{a\alpha ^{n}}\left( \dfrac{1}{2\delta ^{k}}+\dfrac{2|b|}{\alpha ^{n/2}}+\dfrac{2|b|}{\alpha ^{m/2}}\right) <\dfrac{1.5}{\alpha ^{n}}. \end{aligned}$$

Thus, we have

24 $$\begin{aligned} |\delta ^{k}(2a)^{-1}\alpha ^{-n}(1+\alpha ^{m-n})^{-1}-1|<\dfrac{1.5}{\alpha ^{n}}. \end{aligned}$$

Put

$$\begin{aligned} \Lambda _1:=\delta ^{k}(2a)^{-1}\alpha ^{-n}(1+\alpha ^{m-n})^{-1}-1, \end{aligned}$$

and

$$\begin{aligned} \Gamma _1:=k\log \delta -\log (2a) -n\log \alpha -\log (1+\alpha ^{m-n}). \end{aligned}$$

Since $|\Lambda _1|=|e^{\Gamma _1}-1|<1/2$ for $n\ge 4$ (because $1.5/\alpha ^{4}<1/2$), and the inequality $|y|<2|e^{y}-1|$ holds for all $y\in \left( -1/2, 1/2\right)$, it follows that $e^{|\Gamma _1|}<2$ and so

$$\begin{aligned} |\Gamma _1|<e^{|\Gamma _1|}|e^{\Gamma _1}-1|<\dfrac{3}{\alpha ^{n}}. \end{aligned}$$

Thus, we get that

25 $$\begin{aligned} |k\log \delta -\log (2a) -n\log \alpha -\log (1+\alpha ^{m-n})|<\dfrac{3}{\alpha ^{n}}. \end{aligned}$$

We apply Theorem 3 on the left-hand side of (24) with the data:

$$\begin{aligned}&t:=4, \quad \eta _{1}:=\delta , \quad \eta _2:=2a, \quad \eta _3:=\alpha ,\quad \eta _4: =1+\alpha ^{m-n},\\&\qquad b_1:=k, \quad b_2:=-1, \quad b_3:=-n, \quad b_4:=-1. \end{aligned}$$

Furthermore, we take the number field ${\mathbb {K}}:={\mathbb {Q}}(\sqrt{d}, \alpha )$ which has degree $D_{{\mathbb {K}}}:=6$. Since $\max \{1,k,n\}\le n$, we take $B:=n$. First, we note that the left-hand side of (24) is nonzero, since otherwise,

$$\begin{aligned} \delta ^{k}=2a(\alpha ^{n}+\alpha ^{m}). \end{aligned}$$

The left-hand side belongs to the quadratic field ${\mathbb {Q}}(\sqrt{d})$ while the right-hand side belongs to the cubic field ${\mathbb {Q}}(\alpha )$. These fields only intersect when both sides are rational numbers. Since $\delta ^{k}$ is a positive algebraic integer and a unit, we get that to $\delta ^{k} =1$. Hence, $k=0$, which is a contradiction. Thus, $\Lambda _1\ne 0$ and we can apply Theorem 3.

We have $h(\eta _1)=h(\delta )=(\log \delta )/2$ and $h(\eta _3)=h(\alpha )=(\log \alpha )/3$. Furthermore,

$$\begin{aligned} 2a=\dfrac{2\alpha (\alpha +1)}{2\alpha +3}. \end{aligned}$$

The minimal polynomial of 2*a* is $23x^3-20x-8$ and has roots 2*a*, 2*b*, 2*c*. Since $2|b|=2|c|<1$ (by (14)), then

$$\begin{aligned} h(\eta _2)=h(2a)=\dfrac{1}{3}(\log 23+\log (2a)). \end{aligned}$$

On the other hand,

$$\begin{aligned} h(\eta _4)&=h(1+\alpha ^{m-n})\le h(1)+h(\alpha ^{m-n})+\log 2\\&=(n-m)h(\alpha )+\log 2 = \dfrac{1}{3}(n-m)\log \alpha +\log 2. \end{aligned}$$

Thus, we can take $A_1:=3\log \delta$,

$$\begin{aligned} A_2:=2(\log 23+\log (2a)), \quad A_3:=2\log \alpha , \quad A_4:=2(n-m)\log \alpha +6\log 2. \end{aligned}$$

Now, Theorem 3 tells us that

$$\begin{aligned} \log |\Lambda _1|&> -1.4\times 30^{7}\times 4^{4.5}\times 6^{2}(1+\log 6)(1+\log n)(3\log \delta )\\&\quad \times (2(\log 23+\log (2a))(2\log \alpha )(2(n-m)\log \alpha +6\log 2)\\&>-2.33\times 10^{17}(n-m)(\log n)(\log \delta ). \end{aligned}$$

Comparing the above inequality with (24), we get

$$\begin{aligned} n\log \alpha - \log 1.5 < 2.33\times 10^{17}(n-m)(\log n)(\log \delta ). \end{aligned}$$

Hence, we get that

26 $$\begin{aligned} n<8.30\times 10^{17}(n-m)(\log n)(\log \delta ). \end{aligned}$$

We now return to the Diophantine equation (8) and rewrite it as

$$\begin{aligned} \dfrac{1}{2}\delta ^{k}-a\alpha ^{n} = -\dfrac{1}{2}\sigma ^{k}+e(n)+P_m, \end{aligned}$$

we obtain

27 $$\begin{aligned} |\delta ^{k}(2a)^{-1}\alpha ^{-n}-1|\le \dfrac{1}{a\alpha ^{n-m}}\left( \dfrac{1}{\alpha }+\dfrac{1}{\alpha ^{m+n/2}}+\dfrac{1}{2\delta ^{k}\alpha ^{m}}\right) <\dfrac{2.5}{\alpha ^{n-m}}. \end{aligned}$$

Put

$$\begin{aligned} \Lambda _2:=\delta ^{k}(2a)^{-1}\alpha ^{-n}-1, \quad \Gamma _{2}:=k\log \delta -\log (2a)-n\log \alpha . \end{aligned}$$

We assume for technical reasons that $n-m\ge 10$. Therefore, $|e^{\Lambda _2}-1|<1/2$. It follows that

28 $$\begin{aligned} |k\log \delta -\log (2a)-n\log \alpha |=|\Gamma _2|<e^{|\Lambda _2|}|e^{\Lambda _2}-1|<\dfrac{5}{\alpha ^{n-m}}. \end{aligned}$$

Furthermore, $\Lambda _2\ne 0$ (so $\Gamma _2 \ne 0$), since $\delta ^{k}\not \in {\mathbb {Q}}(\alpha )$ by a previous argument.

We now apply Theorem 3 to the left-hand side of (27) with the data

$$\begin{aligned} t:=3, \quad \eta _1:=\delta , \quad \eta _2:=2a, \quad \eta _3:=\alpha , \quad b_1:=k, \quad b_2:=-1, \quad b_3:=-n. \end{aligned}$$

Thus, we have the same $A_1, \,A_2, \,A_3$, as before. Then, by Theorem 3, we conclude that

$$\begin{aligned} \log |\Lambda |>-9.82\times 10^{14}(\log \delta )(\log n)(\log \alpha ). \end{aligned}$$

By comparing with (27), we get

29 $$\begin{aligned} n-m <9.84\times 10^{14}(\log \delta )(\log n). \end{aligned}$$

This was obtained under the assumption that $$ n-m\ge 10 $$, but if $$ n-m<10$$, then the above inequality also holds as well. We replace $$ n-m $$ in (26) by its upper bound that we obtained in (29) and use the fact that $$ \delta ^{k}\le 2\alpha ^{n+3} $$, to obtain bounds on *n* and *k* in terms of $$ \log n $$ and $$\log \delta $$. We now record what we have proved so far.

#### Lemma 5

*Let* (*k*, *n*, *m*) *be a solution to the Diophantine equation* (8) *with* $3\le m<n$, *then*

30 $$\begin{aligned} \quad k< 2.5\times 10^{32}(\log n)^{2}(\log \delta ) \quad \text {and} \quad n<8.2\times 10^{32}(\log n)^{2}(\log \delta )^{2}. \end{aligned}$$

### Absolute bounds

We recall that $(k,n,m)=(k_i,n_i, m_i)$, where $3\le m_i<n_i$, for $i=1,2$ and $1\le k_1<k_2$. Furthermore, $n_i\ge 4$ for $i=1,2$. We return to (28) and write

$$\begin{aligned} |\Gamma _2^{(i)}|:=|k_i\log \delta - \log (2a) -n_i\log \alpha |<\dfrac{5}{\alpha ^{n_i-m_i}}, \quad \text { for } \quad i=1,2. \end{aligned}$$

We do a suitable cross product between $\Gamma _2^{(1)}, \, \Gamma _2^{(2)}$ and $k_1, k_2$ to eliminate the term involving $\log \delta$ in the above linear forms in logarithms:

31 $$\begin{aligned} |\Gamma _3|:&=|(k_1-k_2)\log (2a)+(k_1n_2-k_2n_1)\log \alpha |=|k_2\Gamma _2^{(1)}-k_1\Gamma _2^{(2)}|\nonumber \\&\le k_2|\Gamma _2^{(1)}|+k_1|\Gamma _2^{(2)}| \le \dfrac{5k_2}{\alpha ^{n_1-m_1}}+\dfrac{5k_1}{\alpha ^{n_2-m_2}}\le \dfrac{10n_2}{\alpha ^{\lambda }}, \end{aligned}$$

where $$\displaystyle {\lambda :=\min _{1\le i\le 2} \{n_i-m_i\}.}$$

We need to find an upper bound for $\lambda$. If $10n_2/\alpha ^{\lambda } > 1/2$, we then get

32 $$\begin{aligned} \lambda< \dfrac{\log (20n_2)}{\log \alpha }<4\log (20n_2). \end{aligned}$$

Otherwise, $$ |\Gamma _3|<1/2 $$, so

33 $$\begin{aligned} |e^{\Gamma _3}-1|=|(2a)^{k_1-k_2}\alpha ^{k_1n_2-k_2n_1}-1|<2|\Gamma _3|<\dfrac{20n_2}{\alpha ^{\lambda }}. \end{aligned}$$

We apply Theorem 3 with the data: $t:=2$, $\eta _1 := 2a$, $\eta _2:= \alpha$, $b_1:=k_1-k_2$, $b_2:=k_1n_2-k_2n_1$. We take the number field ${\mathbb {K}}:={\mathbb {Q}}(\alpha )$ and $D_{{\mathbb {K}}}:=3$. We begin by checking that $e^{\Gamma _3}-1\ne 0$ (so $\Gamma _3\ne 0$). This is true, because $\alpha$ and 2*a* are multiplicatively independent, since $\alpha$ is a unit in the ring of integers ${\mathbb {Q}}(\alpha )$ while the norm of 2*a* is 8/23.

We note that $|k_1-k_2|<k_2<n_2$. Furthermore, from (31), we have

$$\begin{aligned} |k_2n_1-k_1n_2|<(k_2-k_1)\dfrac{|\log (2a)|}{\log \alpha }+\dfrac{10k_2}{\alpha ^{\lambda }\log \alpha }<11k_2<11n_2 \end{aligned}$$

given that $\lambda \ge 1$. Therefore, we can take $B:=11n_2$. By Theorem 3, with the same $A_1:=\log 23$ and $A_2:=\log \alpha$, we have that

$$\begin{aligned} \log |e^{\Gamma _3}-1|>-1.55\times 10^{11}(\log n_2)(\log \alpha ). \end{aligned}$$

By comparing this with (33), we get

34 $$\begin{aligned} \lambda <1.56\times 10^{11}\log n_2. \end{aligned}$$

Note that (34) is a better bound than (32), so (34) always holds. Without loss of generality, we can assume that $\lambda = n_i-m_i$, for $i=1,2$ fixed.

We set $\{i,j\}=\{1,2\}$ and return to (25) to replace $(k,n,m)=(k_i,n_i, m_i)$:

35 $$\begin{aligned} |\Gamma _1^{(i)}|=|k_i\log \delta -\log (2a) -n_i\log \alpha -\log (1+\alpha ^{m_i-n_i})|<\dfrac{3}{\alpha ^{n_i}}, \end{aligned}$$

and also return to (28), with $(k, n,m)=(k_j, n_j, m_j)$:

36 $$\begin{aligned} |\Gamma _2^{(j)}|=|k_j\log \delta -\log (2a)-n_j\log \alpha |<\dfrac{5}{\alpha ^{n_j-m_j}}. \end{aligned}$$

We perform a cross product on (35) and (36) to eliminate the terms on $\log \delta$:

37 $$\begin{aligned} |\Gamma _4|:&=|(k_j-k_i)\log (2a)+(k_jn_i-k_in_j)\log \alpha +k_j\log (1+\alpha ^{m_i-n_i})|\nonumber \\&=|k_i\Gamma _2^{(j)}-k_j\Gamma _1^{(i)}|\le k_i|\Gamma _2^{(j)}|+k_j|\Gamma _1^{(i)}|<\dfrac{5k_i}{\alpha ^{n_j-m_j}}+\dfrac{3k_j}{\alpha ^{n_i}}<\dfrac{8n_2}{\alpha ^{\nu }} \end{aligned}$$

with $\nu :=\min \{n_i, n_j-m_j\}$. As before, we need to find an upper bound on $\nu$. If $8n_2/\alpha ^{\nu }>1/2$, then we get

38 $$\begin{aligned} \nu< \dfrac{\log (16n_2)}{\log \alpha }< 4\log (16n_2). \end{aligned}$$

Otherwise, $|\Gamma _4|<1/2$, so we have

39 $$\begin{aligned} |e^{\Gamma _4}-1|\le 2|\Gamma _4|<\dfrac{16n_2}{\alpha ^{\nu }}. \end{aligned}$$

To apply Theorem 3, first if $e^{\Gamma _4}=1$, we obtain

$$\begin{aligned} (2a)^{k_i-k_j}=\alpha ^{k_jn_i-k_in_j}(1+\alpha ^{-\lambda })^{k_j}. \end{aligned}$$

Since $\alpha$ is a unit, the right-hand side in above is an algebraic integer. This is a contradiction, because $k_1<k_2$ so $k_i-k_j\ne 0$, and neither (2*a*) nor $(2a)^{-1}$ are algebraic integers. Hence, $e^{\Gamma _4}\ne 1$. By assuming that $\nu \ge 100$, we apply Theorem 3 with the data:

$$\begin{aligned}&t:=3, \quad \eta _1:=2a, \quad \eta _2:=\alpha , \quad \eta _3:=1+\alpha ^{-\lambda },\\&\, b_1:=k_j-k_i, \quad b_2:=k_jn_i-k_in_j, \quad b_3:=k_j, \end{aligned}$$

and the inequalities (34) and (39). We get

$$\begin{aligned} \nu =\min \{n_i, n_j-m_j\}<1.14\times 10^{14}\lambda \log n_2<1.78\times 10^{25}(\log n_2)^{2}. \end{aligned}$$

The above inequality also holds when $\nu <100$. Furthermore, it also holds when the inequality (38) holds. Therefore, the above inequality holds in all cases. Note that the case $(i, j) =(2, 1)$ leads to $n_1-m_1\le n_1\le n_2+4$ whereas $(i, j) = (1,2)$ leads to $\nu = \min \{n_1, n_2-m_2\}$. Hence, either the minimum is $n_1$, so

40 $$\begin{aligned} n_1< 1.78\times 10^{25}(\log n_2)^{2}, \end{aligned}$$

or the minimum is $n_j-m_j$ and from the inequality (34), we get that

41 $$\begin{aligned} \max _{1\le j\le 2}\{n_j- m_j\}< 1.78\times 10^{25}(\log n_2)^{2}. \end{aligned}$$

Next, we assume that we are in the case (41). We evaluate (35), for $i=1,2$ and make a suitable cross-product to eliminate the terms involving $\log \delta$:

42 $$\begin{aligned} |\Gamma _5|:&=|(k_2-k_1)\log (2a)+(k_2n_1-k_1n_2)\log \alpha \nonumber \\&\quad +k_2\log (1+\alpha ^{m_1-n_1})-k_1\log (1+\alpha ^{m_2-n_2})|\nonumber \\&=|k_1\Gamma _1^{(2)}-k_2\Gamma _1^{(1)}|\le k_1|\Gamma _1^{(2)}|+k_2|\Gamma _1^{(1)}|<\dfrac{6n_2}{\alpha ^{n_1}}. \end{aligned}$$

In the above inequality, we used the inequality (23) to conclude that $\min \{n_1, n_2\}\ge n_1-4$ as well as the fact that $n_i\ge 4$ for $i=1.2$. Next, we apply a linear form in four logarithms to obtain an upper bound to $n_1$. As in the previous calculations, we pass from (42) to

43 $$\begin{aligned} |e^{\Gamma _5}-1|<\dfrac{12n_2}{\alpha ^{n_1}}, \end{aligned}$$

which is implied by (42) except if $n_1$ is very small, say

44 $$\begin{aligned} n_1\le 4\log (12n_2). \end{aligned}$$

Thus, we assume that (44) does not hold, therefore (43) holds. Then, to apply Theorem 3, we first justify that $e^{\Gamma _5}\ne 1$. Otherwise,

$$\begin{aligned} (2a)^{k_1-k_2}=\alpha ^{k_2n_1-k_1n_2}(1+\alpha ^{n_1-m_1})^{k_2}(1+\alpha ^{n_2-m_2})^{-k_1}. \end{aligned}$$

By the fact that $k_1<k_2$, the norm $\mathbf{N }_{{\mathbb {Q}}(\alpha )/{\mathbb {Q}}} (2a) ={8}/{23}$ and that $\alpha$ is a unit, we have that 23 divides the norm $\mathbf{N }_{{\mathbb {K}}/{\mathbb {Q}}} (1+\alpha ^{n_1-m_1})$. The factorization of the ideal generated by 23 in ${\mathcal {O}}_{{\mathbb {Q}}(\alpha )}$ is $(23)={\mathfrak {p}}_1^2{\mathfrak {p}}_2$, where ${\mathfrak {p}}_1=(23, \,\alpha +13)$ and ${\mathfrak {p}}_2=(23,\, \alpha +20)$. Hence, ${\mathfrak {p}}_2$ divides $\alpha ^{n_1-m_1} + 1$. Given that $\alpha \equiv -20\, (\text {mod}\, {\mathfrak {p}}_2)$, then $(-20)^{n_1-m_1}\equiv -1(\text {mod}\,{\mathfrak {p}}_2)$. Taking the norm $\mathbf{N }_{{\mathbb {Q}}(\alpha )/{\mathbb {Q}}}$, we obtain that $(-20)^{n_1-m_1} \equiv -1\,(\text {mod}\, 23)$. If $n_1-m_1$ is even, $-1$ is a quadratic residue modulo 23 and if $n_1-m_1$ is odd then 20 is a quadratic residue modulo 23. But, neither $-1$ nor 20 are quadratic residues modulo 23. Thus, $e^{\Gamma _{5}}\ne 1$.

Then, we apply Theorem 3 on the left-hand side of the inequality (43) with the data

$$\begin{aligned}&t:=4, \quad \eta _1:=2a, \quad \eta _2:=\alpha ,\quad \eta _3:=1+\alpha ^{m_1-n_1}, \quad \eta _4:=1+\alpha ^{m_2-n_2},\\&\qquad b_1:=k_2-k_1, \quad b_2:=k_2n_1-k_1n_2, \quad b_3:=k_2, \quad b_4:=k_1. \end{aligned}$$

Combining the right-hand side of (43) with the inequalities (34) and (41), Theorem 3 gives

45 $$\begin{aligned} n_1<3.02\times 10^{16}(n_1-m_1)(n_2-m_2)(\log n_2)<8.33\times 10^{52}(\log n_2)^{4}. \end{aligned}$$

In the above, we used the facts that

$$\begin{aligned} \min _{1\le i\le 2}\{n_i-m_i\}<1.56\times 10^{11}\log n_2 \quad \text {and}\quad \max _{1\le i\le 2}\{n_i-m_i\}<1.78\times 10^{25}(\log n_2)^{2}. \end{aligned}$$

This was obtained under the assumption that the inequality (44) does not hold. If (44) holds, then so does (45). Thus, we have that inequality (45) holds provided that inequality (41) holds. Otherwise, inequality (40) holds which is a better bound than (45). Hence, we conclude that (45) holds in all possible cases.

By the inequality (22),

$$\begin{aligned} \log \delta \le k_1\log \delta \le n_1\log \alpha +\log 6 <2.38\times 10^{52}(\log n_2)^{4}. \end{aligned}$$

By substituting this into (30) we get $$n_2<4.64\times 10^{137}(\log n_2)^{10}$$, and then, by Lemma 4, with the data $$ r:=10, \,H:=4.64\times 10^{137}$$ and $$L:=n_2 $$, we get that $$ n_2< 4.87\times 10^{165}$$. This immediately gives that $$ n_1<1.76\times 10^{63} $$.

We record what we have proved.

#### Lemma 6

*Let* $(k_i, n_i, m_i)$ *be a solution to the Diophantine equation* (8), *with* $3\le m_i<n_i$ *for* $i\in \{1,2\}$ *and* $1\le k_1<k_2$, *then*

$$\begin{aligned} \max \{k_1, m_1\}<n_1<1.76\times 10^{63}, \quad \text {and} \quad \max \{k_2, m_2\}<n_2<4.87\times 10^{165}. \end{aligned}$$

## Reducing the bounds for $n_1$ and $n_2$

In this section, we reduce the bounds for $n_1$ and $n_2$ given in Lemma 6 to cases that can be computationally treated. For this, we return to the inequalities for $\Gamma _3$, $\Gamma _4$, and $\Gamma _5$.

### The first reduction

We divide both sides of the inequality (31) by $(k_2-k_1)\log \alpha$. We get that

46 $$\begin{aligned} \left| \dfrac{\log (2a)}{\log \alpha }-\dfrac{k_2n_1-k_1n_2}{k_2-k_1}\right| <\dfrac{36n_2}{\alpha ^{\lambda }(k_2-k_1)} \quad \text {with} \quad \lambda : = \min _{1 \le i \le 2}\{n_i-m_i\}. \end{aligned}$$

We assume that $\lambda \ge 10$. Below we apply Lemma 1. We put $\tau := \log (2a)/\log \alpha$, which is irrational and compute its continued fraction

$$\begin{aligned}{}[a_0, a_1, a_2, \ldots ]=[1, 3, 3, 1, 11, 1, 2, 1, 1, 1, 3, 1, 1, 1, 2, 5, 1, 15, 2, 19, 1, 1, 2, 2, \ldots ], \end{aligned}$$

and its convergents

$$\begin{aligned} \left[ \frac{p_0}{q_0},\frac{p_1}{q_1}, \frac{p_2}{q_2}, \ldots \right] =\left[ 1, \frac{4}{3}, \frac{13}{10}, \frac{17}{13}, \frac{200}{153}, \frac{217}{166}, \frac{634}{485}, \frac{851}{651}, \frac{1485}{1136}, \frac{2336}{1787}, \frac{8493}{6497}, \ldots \right] . \end{aligned}$$

Furthermore, we note that taking $M:=4.87\times 10^{165}$ (by Lemma 6), it follows that

$$\begin{aligned} q_{315}>M>n_2>k_2-k_1 \quad \text {and}\quad a(M):=\max \{a_i:0\le i\le 315\}=a_{282}=2107. \end{aligned}$$

Thus, by Lemma 1, we have that

47 $$\begin{aligned} \left| \tau - \dfrac{k_2n_1-k_1n_2}{k_2-k_1}\right| >\dfrac{1}{2109(k_2-k_1)^{2}}. \end{aligned}$$

Hence, combining the inequalities (46) and (47), we obtain

$$\begin{aligned} \alpha ^{\lambda }<75924n_2(k_2-k_1)<1.75\times 10^{336}, \end{aligned}$$

so $\lambda \le 2714$. This was obtained under the assumption that $\lambda \ge 10$. Otherwise, $\lambda<10<2714$ holds as well.

Now, for each $n_i-m_i = \lambda \in [1, 2714]$, we estimate a lower bound for $|\Gamma _4|$, with

48 $$\begin{aligned} \Gamma _4=(k_j-k_i)\log (2a)+(k_jn_i-k_in_j)\log \alpha +k_j\log (1+\alpha ^{m_i-n_i}) \end{aligned}$$

given in the inequality (37), via the procedure described in Sect. 3.3 (LLL-algorithm). We recall that $\Gamma _4\ne 0$. We apply Lemma 3 with the data:

$$\begin{aligned}&t:=3, \quad \tau _1:=\log (2a), \quad \tau _2:=\log \alpha , \quad \tau _3:=\log (1+\alpha ^{-\lambda }),\\&\qquad x_1:=k_j-k_i, \quad x_2:=k_jn_i-k_in_j, \quad x_3:=k_j. \end{aligned}$$

We set $X:= 5.4\times 10^{166}$ as an upper bound to $|x_i|<11n_2$ for all $i=1, 2, 3$, and $C:=(20X)^{5}$. A computer search using *Mathematica* allows us to conclude, together with the inequality (37), that

$$\begin{aligned} 2\times 10^{-671}<\min _{1\le \lambda \le 2714}|\Gamma _4|<8n_2\alpha ^{-\nu },\quad \text {with} \quad \nu :=\min \{n_i, n_j-m_j\} \end{aligned}$$

which leads to $\nu \le 6760$. As we have noted before, $\nu = n_1$ (so $n_1\le 6760$) or $\nu = n_j-m_j$.

Next, we suppose that $n_j-m_j = \nu \le 6760$. Since $\lambda \le 2714$, we have

$$\begin{aligned} \lambda := \min _{1\le i \le 2}\{n_i-m_i\}\le 2714 \quad \text {and} \quad \chi :=\max _{1\le i \le 2}\{n_i-m_i\}\le 6760. \end{aligned}$$

Now, returning to the inequality (42) which involves

49 $$\begin{aligned} \Gamma _5&:=(k_2-k_1)\log (2a)+(k_2n_1-k_1n_2)\log \alpha \nonumber \\ {}&\quad +k_2\log (1+\alpha ^{m_1-n_1})-k_1\log (1+\alpha ^{m_2-n_2})\ne 0. \end{aligned}$$

We use again the LLL algorithm to estimate the lower bound for $|\Gamma _5|$ and thus, find a bound for $n_1$ that is better than the one given in Lemma 6.

We distinguish the cases $\lambda < \chi$ and $\lambda = \chi$.

### The case $\lambda < \chi$

We take $\lambda \in [1, 2714]$ and $\chi \in [\lambda +1, 6760]$ and apply Lemma 3 with the data: $t:=4$,

$$\begin{aligned}&\tau _1:=\log (2a), \quad \tau _2:= \log \alpha , \quad \tau _3: = \log (1+\alpha ^{m_1-n_1}), \quad \tau _4: = \log (1+\alpha ^{m_2-n_2}), \\&\qquad x_1:=k_2-k_1, \quad x_2:= k_2n_1-k_1n_2, \quad x_3: = k_2, \quad x_4:=-k_1. \end{aligned}$$

We also put $X:=5.4\times 10^{166}$ and $C:=(20X)^{9}$. After a computer search in *Mathematica* together with the inequality (42), we can confirm that

$$\begin{aligned} 8\times 10^{-1342}<\min _{\begin{array}{c} 1\le \lambda \le 2714 \\ \lambda +1\le \chi \le 6760 \end{array}}|\Gamma _5| < 6n_2 \alpha ^{-n_1}. \end{aligned}$$

This leads to the inequality

$$\begin{aligned} \alpha ^{n_1}< 7.5\times 10^{1341}n_2. \end{aligned}$$

Substituting for the bound $n_2$ given in Lemma 6, we get that $n_1\le 12172$.

### The case $\lambda =\chi$

In this case, we have

$$\begin{aligned} \Lambda _5:=(k_2-k_1)(\log (2a)+\log (1+\alpha ^{m_1-n_1}))+(k_2n_1-k_1n_2)\log \alpha \ne 0. \end{aligned}$$

We divide through the inequality (42) by $(k_2-k_1)\log \alpha$ to obtain

50 $$\begin{aligned} \left| \dfrac{\log (2a)+\log (1+\alpha ^{m_1-n_1})}{\log \alpha }-\dfrac{k_2n_1-k_1n_2}{k_2-k_1}\right| <\dfrac{21n_2}{\alpha ^{n_1}(k_2-k_1)}. \end{aligned}$$

We now put

$$\begin{aligned} \tau _{\lambda }:=\dfrac{\log (2a)+\log (1+\alpha ^{-\lambda })}{\log \alpha }, \end{aligned}$$

and compute its continued fractions $[a_0^{(\lambda )}, a_1^{(\lambda )} , a_2^{(\lambda )},\ldots ]$, and its convergents $[p_0^{(\lambda )}/q_0^{(\lambda )}, p_1^{(\lambda )}/q_1^{(\lambda )}, p_2^{(\lambda )}/q_2^{(\lambda )},\ldots ]$, for each $\lambda \in [1, 2714]$. Furthermore, for each case we find an integer $t_{\lambda }$ such that $q_{t_{\lambda }}^{(\lambda )}>M:=4.87\times 10^{165}>n_2>k_2-k_1$ and calculate

$$\begin{aligned} a(M):=\max _{1\le \lambda \le 2714}\left\{ a_{i}^{(\lambda )}: 0 \le i \le t_{\lambda }\right\} . \end{aligned}$$

A computer search in *Mathematica* reveals that for $\lambda = 321$, $t_{\lambda } = 330$ and $i=263$, we have that $a(M) = a_{321}^{(330)}=306269$. Hence, combining the conclusion of Lemma 1 and the inequality (50), we get

$$\begin{aligned} \alpha ^{n_1}< 21\times 306271n_2(k_2-k_1)< 1.525\times 10^{338}, \end{aligned}$$

so $n_1\le 2730$. Hence, we obtain that $n_1\le 12172$ holds in all cases ($\nu =n_1$, $\lambda < \chi$ or $\lambda = \chi$). By the inequality (22), we have that

$$\begin{aligned} \log \delta \le k_1\log \delta \le n_1\log \alpha +\log 6 <3475. \end{aligned}$$

By considering the second inequality in (30), we can conclude that $n_2\le 9.9\times 10^{39}(\log n_2)^2$, which immediately yields $n_2<3.36\times 10^{44}$, by a simple application of Lemma 4. We summarise the first cycle of our reduction process as follows:

$$\begin{aligned} n_1\le 12172 \quad \text {and} \quad n_2\le 3.36\times 10^{44}. \end{aligned}$$

From the above inequalities, we note that the upper bound on $n_2$ represents a very good reduction of the bound given in Lemma 6. Hence, we expect that if we restart our reduction cycle with the new bound on $n_2$, then we get a better bound on $n_1$. Thus, we return to the inequality (46) and take $M:=3.36\times 10^{44}$. A computer search in *Mathematica* reveals that

$$\begin{aligned} q_{88}>M>n_2>k_2-k_1 \quad \text {and} \quad a(M):=\max \{a_i: 0\le i\le 88\}=a_{54} =373, \end{aligned}$$

from which it follows that $\lambda \le 752$. We now return to (48) and we put $X:=3.36\times 10^{44}$ and $C:=(10X)^{5}$ and then apply the LLL algorithm in Lemma 3 to $\lambda \in [1, 752]$. After a computer search, we get

$$\begin{aligned} 5.33\times 10^{-184}<\min _{1\le \lambda \le 752}|\Gamma _4| < 8n_2\alpha ^{-\nu }, \end{aligned}$$

then $\nu \le 1846$. By continuing under the assumption that $n_j-m_j=\nu \le 1846$, we return to (49) and put $X:=3.36\times 10^{44}$, $C:=(10X)^9$ and $M:=3.36\times 10^{44}$ for the case $\lambda <\chi$ and $\lambda = \chi$. After a computer search, we confirm that

$$\begin{aligned} 2\times 10^{-366}< \min _{\begin{array}{c} 1\le \lambda \le 752\\ \lambda +1\le \chi \le 1846 \end{array}}|\Gamma _5|<6n_2\alpha ^{-n_1}, \end{aligned}$$

gives $n_1\le 3318$, and $a(M)=a_{175}^{(205)}=206961$, which leads to $n_1\le 772$. Hence, in both cases $n_1\le 3318$ holds. This gives $n_2\le 5\times 10^{42}$ by a similar procedure as before, and $k_1\le 3125$.

We record what we have proved.

#### Lemma 7

*Let* $(k_i, n_i, m_i)$ *be a solution to the Diophantine equation* (8), *with* $3\le m_i<n_i$ *for* $i=1,2$ *and* $1\le k_1<k_2$, *then*

$$\begin{aligned} m_1<n_1\le 3318, \quad k_1\le 3125, \quad \text {and} \quad n_2\le 5\times 10^{42}. \end{aligned}$$

### The final reduction

Returning to (17) and (19) and using the fact that $(x_1, y_1)$ is the smallest positive solution to the Pell equation (1), we obtain

$$\begin{aligned} x_k&= \dfrac{1}{2}(\delta ^k+\sigma ^k) \quad =\quad \dfrac{1}{2}\left( \left( x_1+y_1\sqrt{d}\right) ^{k}+\left( x_1-y_1\sqrt{d}\right) ^k\right) \\&=\dfrac{1}{2}\left( \left( x_1+\sqrt{x_1^2\mp 1}\right) ^{k}+\left( x_1-\sqrt{x_1^2\mp 1}\right) ^k\right) : = Q^{\pm }_{k}(x_1). \end{aligned}$$

Thus, we return to the Diophantine equation $x_{k_1}=P_{n_1}+P_{m_1}$ and consider the equations

51 $$\begin{aligned} Q^{+}_{k_1}(x_1)=P_{n_1}+P_{m_1} \quad \text {and} \quad Q^{-}_{k_1}(x_1)=P_{n_1}+P_{m_1}, \end{aligned}$$

with $$ k_1\in [1, 3125] $$, $$ m_1\in [3,3318 ] $$ and $$n_1\in [m_1+1, 3318 ] $$.

Besides the trivial case $k_1=1$, with the help of a computer search in *Mathematica* on the above equations in (51), we list the only nontrivial solutions in Table 1. We also note that $3+2\sqrt{2}=(1+\sqrt{2})^2$, so these solutions come from the same Pell equation when $d=2$.

**Table 1:** Solutions to $Q_{k_1}^{\pm }(x_1)=P_{n_1}+P_{m_1}$

$$ Q^{+}_{k_1}(x_1) $$				
$k_1$	$x_1$	$y_1$	*d*	$\delta$
2	2	1	3	$2+\sqrt{3}$
2	3	2	2	$3+2\sqrt{2}$
2	4	1	15	$4+\sqrt{15}$
2	5	2	6	$5+2\sqrt{6}$
2	21	2	110	$21+2\sqrt{110}$
2	22	1	483	$22+\sqrt{483}$
2	47	4	138	$47+4\sqrt{138}$

$$ Q^{-}_{k_1}(x_1) $$				
$k_1$	$x_1$	$y_1$	*d*	$\delta$
2	1	1	2	$1+\sqrt{2}$
2	2	1	5	$2+\sqrt{5}$
2	3	1	10	$3+\sqrt{10}$
2	4	1	17	$4+\sqrt{17}$
2	5	1	26	$5+\sqrt{26}$
2	9	1	82	$9+\sqrt{82}$
2	10	1	101	$10+\sqrt{101}$
2	17	1	290	$17+\sqrt{290}$
2	42	1	1765	$42+\sqrt{1765}$
2	47	1	2210	$47+\sqrt{2210}$
2	63	1	3970	$63+\sqrt{3970}$

From Table 1, we set each $\delta :=\delta _{t}$ for $t=1, 2, \ldots , 17$. We then work on the linear forms in logarithms $\Gamma _1$ and $\Gamma _2$, to reduce the bound on $n_2$ given in Lemma 7. From the inequality (28), for $(k,n,m):=(k_2, n_2, m_2)$, we write

52 $$\begin{aligned} \left| k_2\dfrac{\log \delta _t}{\log \alpha }-n_2+\dfrac{\log (2a)}{\log (\alpha ^{-1})}\right| <\left( \frac{5}{\log \alpha }\right) \alpha ^{-(n_2-m_2)}, \quad \mathrm {for}\quad t=1,2, \ldots , 17. \end{aligned}$$

We put

$$\begin{aligned} \tau _{t}:=\dfrac{\log \delta _t}{\log \alpha }, \qquad \mu _t:=\dfrac{\log (2a)}{\log (\alpha ^{-1})}\qquad \text {and} \quad (A_t, B_t):=\left( \frac{5}{\log \alpha }, \alpha \right) . \end{aligned}$$

We note that $\tau _t$ is transcendental by Gelfond-Schneider Theorem (see [2], Theorem 2.1). Thus, $\tau _t$ is irrational. We can rewrite the inequality (52) as

53 $$\begin{aligned} 0<|k_2\tau _t-n_2+\mu _t|<A_tB_t^{-(n_2-m_2)}, \quad \text {for} \quad t=1, 2, \ldots , 17. \end{aligned}$$

We take $M:= 5\times 10^{42}$ which is the upper bound on $n_2$ according to Lemma 7 and apply Lemma 2 to the inequality (53). As before, for each $\tau _t$ with $t=1, 2, \ldots , 17$, we compute its continued fraction $[a_0^{(t)}, a_1^{(t)}, a_2^{(t)}, \ldots ]$ and its convergents $p_0^{(t)}/q_0^{(t)}, p_1^{(t)}/q_1^{(t)}, p_2^{(t)}/q_2^{(t)}, \ldots$. For each case, by means of a computer search in *Mathematica*, we find an integer $s_{t}$ such that

$$\begin{aligned} q^{(t)}_{s_t}> 3\times 10^{43}=6M \qquad \text { and } \qquad \epsilon _t:=\Vert \mu _t q^{(t)}\Vert -M\Vert \tau _t q^{(t)}\Vert >0. \end{aligned}$$

We finally compute all the values of $b_t:=\lfloor \log (A_t q^{(t)}_{s_t}/\epsilon _t)/\log B_t \rfloor$. The values of $b_t$ correspond to the upper bounds on $n_2-m_2$, for each $t=1, 2, \ldots , 17$, according to Lemma 2. The results of the computation for each *t* are recorded in Table 2.

**Table 2:** First reduction computation results

*t*	$$\delta _t$$	$$s_t$$	$$q_{s_t} $$	$$\epsilon _t>$$	$$b_t$$
1	$2+\sqrt{3}$	85	$8.93366\times 10^{43}$	0.3100	374
2	$4+\sqrt{15}$	90	$3.90052\times 10^{43}$	0.3124	371
3	$5+2\sqrt{6}$	80	$3.16032\times 10^{43}$	0.0122	382
4	$21+2\sqrt{110}$	88	$6.33080\times 10^{43}$	0.2200	374
5	$22+\sqrt{483}$	75	$4.19689\times 10^{43}$	0.2361	372
6	$47+4\sqrt{138}$	96	$7.76442\times 10^{43}$	0.3732	373
7	$1+\sqrt{2}$	78	$1.46195 \times 10^{44}$	0.3328	375
8	$2+\sqrt{5}$	94	$1.48837\times 10^{44}$	0.2146	377
9	$3+\sqrt{10}$	88	$4.21425\times 10^{43}$	0.1347	374
10	$4+\sqrt{17}$	92	$1.11753\times 10^{44}$	0.2529	375
11	$5+\sqrt{26}$	98	$3.23107\times 10^{43}$	0.1043	374
12	$9+\sqrt{82}$	74	$5.25207\times 10^{43}$	0.2181	373
13	$10+\sqrt{101}$	94	$1.86122\times 10^{44}$	0.2672	377
14	$17+\sqrt{290}$	87	$1.06422\times 10^{44}$	0.0193	384
15	$42+\sqrt{1765}$	78	$3.81406\times 10^{43}$	0.1768	373
16	$47+\sqrt{2210}$	94	$3.92482\times 10^{43}$	0.4476	370
17	$63+\sqrt{3970}$	85	$6.00550\times 10^{43}$	0.4056	371

By replacing $(k, n, m):=(k_2, n_2, m_2)$ in the inequality (25), we can write

54 $$\begin{aligned} \left| k_2\dfrac{\log \delta _t}{\log \alpha }-n_2+\dfrac{\log (2a(1+\alpha ^{-(n_2-m_2)}))}{\log (\alpha ^{-1})}\right| < \left( \dfrac{3}{\log \alpha }\right) \alpha ^{-n_2}, \quad \mathrm {for} \quad t=1,2,\ldots , 17. \end{aligned}$$

We now put

$$\begin{aligned} \tau _{t}:=\dfrac{\log \delta _t}{\log \alpha }, \quad \mu _{t, n_2-m_2}:=\dfrac{\log (2a(1+\alpha ^{-(n_2-m_2)}))}{\log (\alpha ^{-1})}\quad \text {and} \quad (A_t, B_t):=\left( \frac{3}{\log \alpha }, \alpha \right) . \end{aligned}$$

With the above notations, we can rewrite the inequality (54) as

55 $$\begin{aligned} 0<|k_2\tau _t - n_2+\mu _{t, n_2-m_2}|<A_tB_t^{-n_2}, \quad \mathrm {for} \quad t=1,2, \ldots , 17. \end{aligned}$$

We again apply Lemma 2 to the inequality (55), for

$$\begin{aligned} t=1, 2, \ldots , 17, \quad n_2-m_2 =1, 2, \ldots , b_t, \quad \text {with}\quad M:=5\times 10^{43}. \end{aligned}$$

We take

$$\begin{aligned} \epsilon _{t, n_2-m_2}:=\Vert \mu _t q^{(t, n_2-m_2)}\Vert -M\Vert \tau _t q^{(t, n_2-m_2)}\Vert >0, \end{aligned}$$

and

$$\begin{aligned} b_t=b_{t, n_2-m_2}:=\lfloor \log (A_t q^{(t, n_2-m_2)}_{s_t}/\epsilon _{t, n_2-m_2})/\log B_t \rfloor . \end{aligned}$$

With the help of *Mathematica*, we obtain the results in Table 3.

**Table 3:** Final reduction computation results

*t*	1	2	3	4	5	6	7	8	9
$b_{t, n_2-m_2}$	388	389	394	394	393	394	396	392	392

*t*	10	11	12	13	14	15	16	17	
$b_{t, n_2-m_2}$	396	392	408	390	396	396	388	389	

Thus, $\max \{b_{t, n_2-m_2}: t=1, 2, \ldots , 17$ and $n_2-m_2 = 1, 2, \ldots b_t\} \le 408$. So, by Lemma 2, we have that $n_2\le 408$, for all $t=1,2, \ldots , 17$, and by the inequality (23) we have that $n_1\le n_2+4$. From the fact that $\delta ^{k}\le 2\alpha ^{n+3}$, we can conclude that $k_1< k_2\le 133$. Collecting everything together, our problem is reduced to search for the solutions for (21) in the following range

$$\begin{aligned} 1\le k_1<k_2\le 133, \quad 0\le m_1<n_1 \in [3, 408], \quad \text {and} \quad 0\le m_2<n_2 \in [3, 408]. \end{aligned}$$

After a computer search for the solutions to the Diophantine equations in (21) on the range above, we obtained the following solutions, which are the only solutions for the exceptional *d* cases we have stated in Theorem 1:

For the $+1$ case:

$$\begin{aligned} (d=2)\,&x_1 =3=P_6+P_0=P_5+P_3, \quad x_2=17=P_{12}+P_3;\\ (d=3)\,&x_1 =2=P_3+P_0=P_3+P_3,\, x_2=7=P_9+P_0=P_7+P_6,\, \\&x_3=26=P_{13}+P_8;\\ (d=6)\,&x_1 =5=P_8+P_0 = P_7+P_3= P_6+P_5,\\&x_2=49=P_{16}+P_0=P_{15}+P_{12}=P_{14}+P_{13}; \\ (d=15)\,&x_1=4=P_7+P_0=P_6+P_3=P_5+P_5, \quad x_2=31=P_{14}+P_6;\\ (d=110)\,&x_1=21=P_{13}+P_0=P_{12}+P_{8}=P_{11}+P_{10}, \\&x_2=881=P_{26}+P_{17}=P_{25}+P_{22};\\ (d=483)\,&x_1=22=P_{13}+P_{3}, \quad x_2=967=P_{26}+P_{20}=P_{25}+P_{23}. \end{aligned}$$

For the $-1$ case:

$$\begin{aligned} (d=2)\,&x_1=1=P_3+P_0, \quad x_2=7=P_9+P_0=P_8+P_5=P_7+P_6,\\&x_3=41=P_{15}+P_7=P_{14}+P_{10}=P_{13}+P_{12};\\ (d=5)\,&x_1=2=P_5+P_0=P_3+P_3, \quad x_2=38=P_{15}+P_{3};\\ (d=10)\,&x_1=3=P_6+P_0 = P_5+P_3, \quad x_2=117=P_{19}+P_{6};\\ (d=17)\,&x_1=4=P_7+P_0=P_6+P_3=P_5+P_5,\quad x_2=P_{22}+P_{6}. \end{aligned}$$

This completes the proof of Theorem 1.

## Proof of Theorem 2

The proof of Theorem 2 follows from similar steps, techniques, and arguments as given in the proof of Theorem 1. So, we do not give the details here. Below, we give the solutions to the Diophantine equation (9) for the exceptional *d* cases stated in Theorem 2.

For the $+4$ case:

$$\begin{aligned} (d=3)\,&X_1=4=P_7+P_0=P_6+P_3=P_5+P_5, \\&X_2=14=P_{11}+P_5=P_{10}+P_8,\quad X_3=52=P_{16}+P_6;\\ (d=5)\,&X_1=3=P_6+P_0 = P_5+P_3, \quad X_2=7=P_9+P_0=P_7+P_6,\, \\&X_3=18=P_{12}+P_5;\\ (d=21)\,&X_1 =5=P_8+P_0 = P_7+P_3= P_6+P_5,\\&X_2=23=P_{13}+P_5=P_{12}+P_{9}, \quad X_3=2525=P_{30}+P_{11}. \end{aligned}$$

For the $-4$ case:

$$\begin{aligned} (d=2)\,&X_1=2=P_5+P_0=P_3+P_3, \quad X_2=14=P_{11}+P_5=P_{10}+P_{8};\\ (d=5)\,&X_1=1=P_3+P_0, \quad X_2=4=P_7+P_0=P_6+P_3=P_5+P_5, \\&X_3=11=P_{10}+P_5=P_{9}+P_7, \quad X_4=29=P_{14}+P_{3}. \end{aligned}$$
